# Psychosomatic symptoms during South East Asian haze crisis are related to changes in cerebral hemodynamics

**DOI:** 10.1371/journal.pone.0208724

**Published:** 2019-01-07

**Authors:** Benjamin Y. Tan, Adriel Z. Leong, Aloysius S. Leow, Nicholas J. Ngiam, Bridget S. Ng, Manasi Sharma, Leonard L. Yeo, Philip A. Seow, Chiew S. Hong, Young H. Chee, Jintao Chen, Zhengdao Du, Lily Y. Wong, Amit Batra, Nabin Sarkar, Hock-Luen Teoh, Roger C. Ho, Vijay K. Sharma

**Affiliations:** 1 Division of Neurology, Department of Medicine, National University Health System, Singapore; 2 Yong Loo Lin School of Medicine, National University of Singapore, Singapore; 3 Department of Psychological Medicine, National University Health System, Singapore; Universita degli Studi di Padova, ITALY

## Abstract

**Objectives:**

Forest fires in South Asia lead to widespread haze, where many healthy individuals develop psychosomatic symptoms. We investigated the effects of haze exposure on cerebral hemodynamics and new symptoms. We hypothesised that vasoactive substances present in the haze, would lead to vasodilation of cerebral vasculature, thereby altering cerebral hemodynamics, which in turn may account for new psychosomatic symptoms.

**Methods:**

Seventy-four healthy volunteers were recruited, and serial transcranial Doppler (TCD) ultrasonography was performed to record blood flow parameters of bilateral middle cerebral arteries (MCA). The first TCD was performed in an air-conditioned environment. It was repeated outdoors after the participants spent 30-minutes in the haze environment. The prevailing level of pollutant standards index (PSI) was recorded. Appropriate statistical analyses were performed to compare cerebral hemodynamics at baseline and after haze exposure in all participants. Subgroup analyses were then employed to compare the findings between symptomatic and asymptomatic participants.

**Results:**

Study participants’ median age was 30 years (IQR 26–34), and new psychosomatic symptoms were reported by 35 (47.3%). There was a modest but significant decrease in pulsatility index (PI) and resistivity index (RI) in the left MCA after haze exposure (PI: p = 0.026; RI: p = 0.021). When compared to baseline parameters, haze exposure resulted in significantly lower mean PI (p = 0.001) and RI (p = 0.001) in symptomatic patients, but this difference was not present in asymptomatic patients (PI: p = 0.919; RI: p = 0.970).

**Conclusion:**

Haze causes significant alterations in cerebral hemodynamics in susceptible individuals, probably responsible for various psychosomatic symptoms. The prognostic implications and health effects of haze require evaluation in a larger study.

## Introduction

Forest fires from farmers who use the ‘slash-and-burn’ technique for clearing land for agriculture result in seasonal haze that affects large parts of Southeast Asia for several months of the year.[1] The commonly afflicted countries include Singapore, Malaysia, Thailand and Indonesia. These forest fires have been of serious concern in view of their socioeconomic and political impact.[2] During the severe haze crises in 1997 and 2006, estimated economic losses attributable to the haze were US$9 million and US$50 million, respectively.[3, 4] These were largely due to decreased tourism, reduced efficiency in manufacturing and construction (industries involving largely outdoor work), and reduced human productivity (increased sick leave).[5]

Besides socioeconomic and political concerns, the health impact of the haze is also of equal importance. While long-term exposure to air pollution is known to have adverse impact on population health, the effects of short-term exposure to season haze (which only occurs for a few months in a year and has different contents than usual pollution) have not been clearly established. A previous study demonstrated negative impacts of the seasonal haze, which included increased frequency of emergency attendances for respiratory symptoms such as cough, sore throat and acute exacerbations of chronic obstructive airway disease.[6] Interestingly, extent of the air pollution correlated with the severity and frequency of symptoms experienced by the exposed population.[7, 8]

While the respiratory symptoms and irritation in eyes may be reported by the population exposed to higher concentrations of air pollutants, mechanisms for various non-specific symptoms like headache, lethargy, insomnia and other psychological symptoms are difficult to explain. Some studies postulated these symptoms to be related to changes in blood pressure and cerebral hemodynamics.[9, 10] Perceived dangerous pollutant standards index (PSI) values by susceptible individuals is also associated with negative psychological stress and symptoms.[11–13] In this opportunistic study, we investigated the effect of acute short-term haze exposure on cerebral hemodynamics in healthy individuals, in addition to the relationship between haze exposure and various psychosomatic symptoms. Psychosomatic symptoms were defined as physical and psychological symptoms commonly encountered during a haze crisis as previously studied by The Southeast Asian Haze Research Consortium.[11] We hypothesised that vasoactive substances present in the haze would cause vasodilation of cerebral vasculature and the altered cerebral hemodynamics might result in various psychosomatic symptoms.

## Methods

This study was conducted on healthy volunteers during a phase of ‘seasonal haze’ in Singapore during 2015. We excluded the participants who were older than 55 years of age to minimize the potential confounding effect of co-existing atherosclerosis or undetected cerebrovascular disease on their cerebral hemodynamics.[14] Baseline demographic and presence of previous medical conditions (hypertension, diabetes mellitus, hypercholesterolemia and migraine) were recorded. Two brachial artery blood pressure recordings were obtained from each arm using an automated cuff while participants were seated comfortably, and the higher reading was used for the systolic blood pressure, diastolic blood pressure and heart rate. Any new psychosomatic symptoms experienced by the subjects after 30 minutes of haze exposure were also captured using an anonymized standardized questionnaire, which included commonly experienced symptoms during the haze (sore throat, nausea, anxiety, insomnia, poor appetite, headache, neck stiffness, cough, sputum, breathlessness, runny nose, joint pain, rash, lethargy, itching and watery eyes). End-tidal CO2 (EtCO2) was measured with a capnometer (TIDAL Wave Sp Model 715; Novametrix, Wallingford, CT). All participants were asked to breathe through a plastic tube at the time of testing (beginning and at the end of breath holding test). The capnometer was used for the measurement of EtCO2 in both indoor and outdoor haze air.

All study participants were living in Singapore and working in the Western region of Singapore during the conduct of the study. They had not travelled abroad in the preceding 2 weeks, and had a minimum exposure of 2 weeks of ‘seasonal haze’ before participating in the study.

Written informed consent was obtained from all participants prior to the enrolment. Ethics approval was obtained from the Domain Specific Review Board (DSRB), Office of Human Research Protection Programme, National Healthcare Group (NHG), prior to the initiation of this study (NHG/DSRB Reference number 2014/00602). This study was performed in accordance with the guidelines of the Declaration of Helsinki and Belmont Report.

### Transcranial Doppler (TCD)

Cerebral hemodynamics was evaluated with Digi-lite (Rimed Ltd, Israel) TCD system, which employs 2-MHz pulsed wave ultrasound to evaluate the blood flow characteristics in major intracranial arteries. TCD studies were performed by experienced sonographers, credentialed by the American Society of Neuroimaging. We obtained the optimal blood flow parameters from bilateral middle cerebral arteries (MCA), which was defined as the strongest flow signal obtained at a depth of 45–60 mm (sample volume 8mm).[15] Other major intracranial arteries were excluded, to minimise the time spent during the evaluation, specifically during outdoors in haze conditions. The recorded TCD parameters included peak systolic velocity (PSV), end-diastolic velocity (EDV) and mean flow velocity (MFV) in centimetres per second (cm/s). Cerebrovascular resistance index (CVRi) (mmHg/cms^-1^) was calculated by mean arterial pressure (mmHg) / mean flow velocity (cm/s) whereby mean arterial pressure = 2/3 × diastolic blood pressure + 1/3 × systolic blood pressure.[10] Pulsatility index (PI) was calculated for each side as peak systolic velocity—end diastolic velocity) / mean flow velocity.[15] PI represents the flow characteristics in the insonated artery.[16] Resistivity index (RI), which is calculated by (peak systolic velocity—end diastolic velocity) / peak systolic velocity was also calculated.[17] Being ratios, PI and RI are not affected by sonographers’ technical experience or angle of TCD insonation. Breath-holding index (BHI) represents the vasodilatory reserve in the vascular bed supplied by the index artery.[18] It was calculated for bilateral MCAs in all study participants, according to a validated standardised method at our neurovascular laboratory.[19] Briefly, the Probe Holder LMY-3 (Rimed Ltd, Israel) was used to fix the TCD monitoring probes for bilateral MCA. Subjects were asked to hold breath for 30 seconds and MFVs in the MCAs were monitored. BHI was calculated as [(MFVend of breath holding—MFV_baseline_)/MFV_baseline_) x 100/number of seconds of breath-holding].[18] A value of >0.69 represented normal BHI. All TCD evaluations were repeated outdoors after the study participants had spent at least 30 minutes in the haze environment.

### Pollutant standards index (PSI)

PSI was developed by the United States Environmental Protection Agency to describe the air quality. It was measured using five pollutants (carbon monoxide, sulfur dioxide, nitrogen dioxide, ozone and fine particles <10micrometers (PM10)).[20] It ranges from 0–500 and is grades the air quality as good (0–50), moderate (51–100), unhealthy (101–200), very unhealthy (201–300), and hazardous for health (>300).[20, 21] The ambient air in Singapore is monitored through a comprehensive network of air monitoring stations located in different regions of Singapore, which is administered by the National Environment Agency (NEA).[22] The PSI for the Western region of Singapore was monitored and released by NEA on an hourly basis during the study period. The prevailing region-specific PSI reading at the point of haze exposure was recorded by our study investigators to perform further statistical analyses.

### Statistical analyses

Continuous variables were summarized as means ±SD or medians (and interquartile ranges), and categorical variables as proportions. PSI level experienced between symptomatic and asymptomatic participants were analysed by independent t-test while EtCO2 levels in indoor and outdoor haze air were analysed with paired t-test. The effects of haze exposure and presence of symptoms in study participants (1 or more new symptoms that started with haze exposure) on cerebral hemodynamics were analysed with the use of two-way repeated measures analysis of variance (ANOVA). Post hoc test was carried out for significant interactions. We also employed a linear regression to evaluate the correlation of mean PI and RI with the number of new symptoms experienced and the PSI value, quantified by Pearson’s correlation coefficient (r). Mean PI or RI were obtained by calculating the mean of the left and right MCA corresponding values. A p-value of less than 0.05 was considered as statistically significant. All data was analysed using IBM SPSS Statistics version 20.

## Results

Seventy-four healthy volunteers were enrolled in this study. The median age was relatively young (30 years, IQR 26–34). Most of the subjects were Chinese, while the rest were Indian, Malay, Filipino or from other ethnicities. Owing to the young population, the prevalence of medical comorbidities including hypertension, hypercholesterolemia and diabetes mellitus were expectedly low in our study (Table 1). In our study population, 35 (47.3%) participants developed new symptoms after haze exposure. Headache and lethargy were the most common symptoms encountered.

**Table 1:** Baseline characteristics of study participants (n = 74).

Characteristic	
Median age, years (IQR)	30 (26–34)
Female sex, n (%)	45 (60.8%)
Ethnicity, n (%)	
Chinese	43 (58.1%)
Indian	12 (16.2%)
Malay	5 (6.8%)
Filipino	11 (14.9%)
Others	3 (4.1%)
Hypertension, n (%)	4 (5.4%)
Hypercholesterolemia, n (%)	3 (4.1%)
Diabetes Mellitus, n (%)	3 (4.1%)
Smoker, n (%)	4 (5.4%)
Migraine, n (%)	8 (10.8%)

The baseline EtCO2 in outdoor haze air (38.1±0.9 mmHg) was higher than in indoor air (35.8±0.7 mmHg, p<0.001). Mean outdoor haze PSI level experienced by our study participants was 116±39 units. While 36 (48.6%) were exposed to the moderate range (51–100), 38 (51.4%) participants experienced haze in the unhealthy range (PSI 101–200). In the asymptomatic group, 20 participants (51.3%) were exposed to haze in the moderate range, while 19 participants (48.7%) experienced haze in the unhealthy range. In the symptomatic group, 16 participants (45.7%) were exposed to haze in the moderate range, while 19 participants (54.3%) experienced haze in the unhealthy range. Overall, there was no significant difference (p = 0.729) in PSI level experienced between symptomatic (118±40 units) and asymptomatic participants (115±39 units).

### Effects of haze exposure on cerebral hemodynamics

The effect of outdoor haze exposure and presence of symptoms experienced by participants on TCD findings and vital signs are shown in Table 2. There was a statistically significant effect of haze exposure on PI and RI values in the left MCA (PI: p = 0.026; RI: p = 0.021) and the mean MCA (PI: p = 0.021; RI, p = 0.015), but only RI value in the right MCA (RI, p = 0.046). There were no significant differences in PSV, EDV, MFV, CVRi or BHI observed after haze exposure in bilateral MCAs (Table 2).

**Table 2:** ANOVA repeated measures analysis (n = 74) stratifying groups into asymptomatic (n = 39) and symptomatic (n = 35) volunteers.

Haemodynamic Parameter	Baseline	After Haze Exposure	Mean difference (95% CI)	Haze Exposure (within group)		Symptoms (between group)		Haze*Symptoms		Post Hoc
				*F*	*p*	*F*	*p*	*F*	*p*	*p*
Left MCA										
Mean flow velocity, cm/s (mean, SD)										
Asymptomatic	61.8 (11.5)	60.0 (9.9)	-1.8 (-4.8–1.2)	0.005	0.942	3.544	0.064	2.230	0.140	N/A
Symptomatic	64.8 (13.0)	66.5 (13.6)	1.7 (2.1–5.4)							
Peak systolic velocity, cm/s (mean, SD)										
Asymptomatic	100.0 (19.4)	97.1 (16.3)	-2.9 (-7.8–2.1)	0.726	0.397	1.933	0.169	0.644	0.425	N/A
Symptomatic	104.3 (20.4)	104.2 (20.4)	-0.09 (-5.1–4.9)							
End diastolic velocity, cm/s (mean, SD)										
Asymptomatic	42.7 (7.9)	41.4 (6.9)	-1.3 (-3.6–1.0)	0.649	0.423	6.527	**0.013**	4.583	**0.036**	0.335
Symptomatic	45.3 (9.8)	48.2 (10.9)	2.9 (-0.5–6.2)							**0.046**
Cerebrovascular resistance index (mmHg/cms^-1^)										
Asymptomatic	1.47 (0.33)	1.52 (0.30)	0.05 (-0.05–0.14)	0.436	0.511	0.391	0.534	0.604	0.440	N/A
Symptomatic	1.45 (0.33)	1.44 (0.41)	-0.004 (-0.10–0.09)							
Pulsatility index										
Asymptomatic	0.93 (0.08)	0.93 (0.07)	0.004 (-0.03–0.04)	5.138	**0.026**	8.326	**0.005**	6.518	**0.013**	0.836
Symptomatic	0.91 (0.10)	0.85 (0.10)	-0.06 (-0.11 –-0.02)							**0.001**
Resistivity index										
Asymptomatic	0.57 (0.03)	0.57 (0.03)	0.002 (-0.01–0.01)	5.612	**0.021**	11.13	**0.001**	7.065	**0.010**	0.834
Symptomatic	0.57 (0.04)	0.54 (0.04)	-0.03 (-0.04 –-0.01)							**0.001**
Breath holding index										
Asymptomatic	1.6 (0.6)	1.6 (0.6)	-0.05 (-0.25–0.14)	0.000	0.983	0.093	0.761	0.596	0.443	N/A
Symptomatic	1.6 (0.5)	1.7 (0.5)	0.05 (-0.15–0.25)							
Right MCA										
Mean flow velocity, cm/s (mean, SD)										
Asymptomatic	59.3 (9.9)	60.7 (11.4)	1.4 (-2.4–5.1)	1.922	0.170	1.453	0.232	0.089	0.767	N/A
Symptomatic	61.8 (13.1)	63.9 (12.3)	2.1 (-1.3–5.6)							
Peak systolic velocity, cm/s (mean, SD)										
Asymptomatic	96.6 (17.0)	98.6 (17.3)	1.9 (-3.5–7.4)	0.615	0.435	0.104	0.748	0.074	0.786	N/A
Symptomatic	98.4 (20.6)	99.3 (18.9)	0.94 (-4.1–6.0)							
End diastolic velocity, cm/s (mean, SD)										
Asymptomatic	40.6 (6.6)	41.8 (8.6)	1.2 (-1.6–4.0)	3.292	0.074	4.291	**0.042**	0.453	0.503	N/A
Symptomatic	43.5 (9.7)	46.1 (9.6)	2.6 (-0.6–5.7)							
Cerebrovascular resistance index (mmHg/cms^-1^)										
Asymptomatic	1.51 (0.28)	1.51 (0.30)	-0.01 (-0.10–0.09)	0.931	0.338	0.005	0.943	0.557	0.458	N/A
Symptomatic	1.55 (0.46)	1.49 (0.34)	-0.06 (-0.17–0.05)							
Pulsatility index										
Asymptomatic	0.94 (0.07)	0.94 (0.08)	0.00 (-0.03–0.03)	3.520	0.065	19.71	**<0.001**	3.404	0.069	N/A
Symptomatic	0.89 (0.10)	0.84 (0.13)	-0.05 (-0.10–-0.003)							
Resistivity index										
Asymptomatic	0.58 (0.03)	0.58 (0.03)	0.00 (-0.01–0.01)	4.141	**0.046**	19.44	**<0.001**	3.424	0.068	N/A
Symptomatic	0.56 (0.04)	0.53 (0.05)	-0.02 (-0.04 –-0.002)							
Breath holding index										
Asymptomatic	1.6 (0.4)	1.5 (0.5)	-0.07 (-0.22–0.09)	0.346	0.558	1.755	0.189	0.293	0.590	N/A
Symptomatic	1.7 (0.6)	1.7 (0.5)	-0.003 (-0.20–0.19)							
Mean MCA										
Mean flow velocity, cm/s (mean, SD)										
Asymptomatic	60.5 (9.4)	60.3 (9.3)	0.23 (-2.2–2.7)	0.783	0.379	2.780	0.100	1.263	0.265	N/A
Symptomatic	63.3 (11.9)	65.2 (12.2)	-1.9 (-4.9–1.1)							
Peak systolic velocity, cm/s (mean, SD)										
Asymptomatic	98.3 (16.2)	97.8 (14.8)	0.46 (-3.2–4.2)	0.000	0.990	0.873	0.353	0.118	0.733	N/A
Symptomatic	101.3 (18.6)	101.8 (18.2)	-0.4 (-4.2–3.3)							
End diastolic velocity, cm/s (mean, SD)										
Asymptomatic	41.7 (6.2)	41.6 (6.9)	0.06 (-1.8–2.0)	2.522	0.117	6.217	**0.015**	2.770	0.100	N/A
Symptomatic	44.4 (8.9)	47.1 (9.7)	-2.7 (-5.6–0.17)							
Cerebrovascular resistance index (mmHg/cms^-1^)										
Asymptomatic	1.49 (0.27)	1.51 (0.27)	0.02 (-0.05–0.09)	0.060	0.808	0.087	0.769	1.170	0.283	N/A
Symptomatic	1.50 (0.36)	1.47 (0.35)	-0.03 (-0.10–0.04)							
Pulsatility index										
Asymptomatic	0.93 (0.06)	0.94 (0.07)	0.002 (-0.03–0.03)	5.568	**0.021**	16.66	**<0.001**	6.250	**0.015**	0.919
Symptomatic	0.90 (0.10)	0.84 (0.10)	-0.06 (-0.10 –-0.02)							**0.001**
Resistivity index										
Asymptomatic	0.57 (0.02)	0.57 (0.03)	0.00 (-0.01–0.01)	6.196	**0.015**	18.22	**<0.001**	6.453	**0.013**	0.970
Symptomatic	0.56 (0.04)	0.54 (0.04)	0.02 (-0.04 –-0.01)							**0.001**
Breath holding index										
Asymptomatic	1.60 (0.47)	1.54 (0.48)	0.06 (-0.07–0.20)	0.149	0.701	0.762	0.386	0.785	0.379	N/A
Symptomatic	1.64 (0.49)	1.66 (0.41)	-0.02 (-0.17–0.12)							
Vital Signs										
Systolic blood pressure, mmHg (mean, SD)										
Asymptomatic	118.6 (11.0)	117.6 (10.8)	-0.97 (-4.2–2.3)	0.917	0.341	1.811	0.183	0.004	0.949	N/A
Symptomatic	121.8 (10.2)	120.7 (12.5)	-1.1 (-4.0–1.9)							
Diastolic blood pressure, mmHg (mean, SD)										
Asymptomatic	72.0 (7.3)	74.0 (6.6)	2.0 (-0.3–4.3)	6.066	**0.016**	2.427	0.124	0.027	0.870	N/A
Symptomatic	74.5 (9.6)	76.8 (9.7)	2.3 (-0.4–5.0)							
Heart rate, beats per minute (mean, SD)										
Asymptomatic	76.4 (14.5)	79.4 (13.2)	3.0 (-0.4–6.4)	5.427	**0.023**	0.139	0.710	0.048	0.828	N/A
Symptomatic	77.6 (8.1)	80.1 (11.6)	2.5 (-0.8–5.8)							

In terms of changes in the vital signs after haze exposure, the effect of haze exposure was significant on diastolic blood pressure (p = 0.016) and heart rate (p = 0.023), but the systolic blood pressure remained similar (p = 0.341) (Table 2).

### Subgroup analyses—Asymptomatic and symptomatic groups

Subgroup analyses showed no significant differences in PSV, MFV, CVRi or BHI within and between the symptomatic and asymptomatic groups (Table 2). However, there were statistically significant interactions between the effects of haze exposure and presence of symptoms on EDV (p = 0.036), PI (p = 0.013) and RI (p = 0.010) values in the left MCA. These significant interactions were not replicated in the right MCA. Significant interactions were also found on PI and RI in mean MCA (mean MCA PI: p = 0.015; and mean MCA RI: p = 0.013). All study participants were young individuals who did not suffer from any pulmonary disease. EtCO2 increased equally in both symptomatic and asymptomatic subjects. Therefore, it was not included in ANOVA and post hoc tests.

Results of post hoc test performed for significant interactions were reported (Table 2). Of note, haze exposure resulted in significantly lower mean PI (p = 0.001) and RI (p = 0.001) values in symptomatic patients, but this difference was not present in asymptomatic patients (PI: p = 0.919; RI: p = 0.970) (S1 and S2 Figs).

### Correlating PI and RI with symptoms

**Fig 1:**
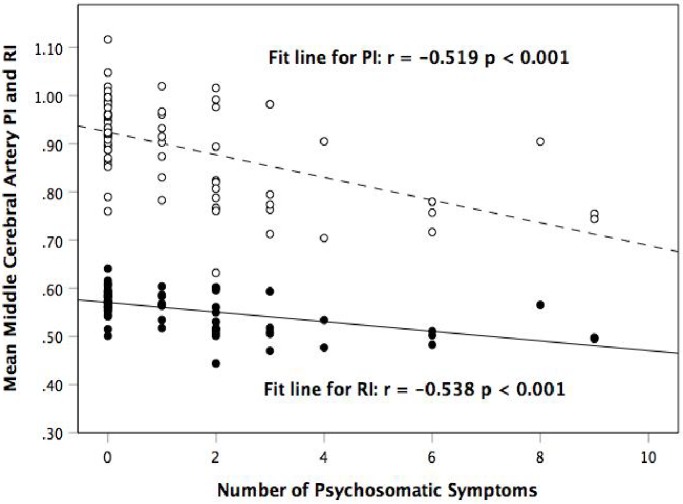
Scatter plot showing the correlation between mean middle cerebral artery pulsatility index (PI) and resistivity index (RI), and number of psychosomatic symptoms.

We demonstrated a negative correlation between mean MCA PI and the number of psychosomatic symptoms, where the higher the number of psychosomatic symptoms experienced, the lower the mean MCA PI (r = -0.519, p<0.001). A similar relationship was observed when studying the correlation between mean MCA RI and the number of psychosomatic symptoms participants had experienced (r = -0.538, p<0.001) (Fig 1).

### Correlating PI and RI with PSI

**Fig 2:**
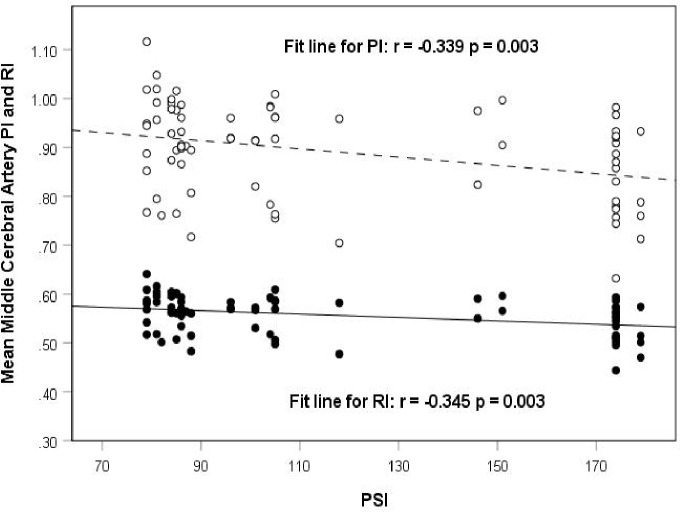
Scatter plot showing the correlation between mean middle cerebral artery pulsatility index (PI) and resistivity index (RI), and pollutant standards index (PSI).

A statistically significant negative correlation was found when evaluating both mean MCA PI and RI with PSI levels (PI: r = -0.339, p = 0.003; RI: r = -0.345, p = 0.003) (Fig 2).

## Discussion

The main findings of our study are: (1) There was a modest but significant decrease in PI and RI in the left MCA after haze exposure, along with notable decreases in PI and RI after haze exposure in the right MCA showing a trend towards significance but, perhaps because of a small sample size, did not achieve statistical significance; (2) On subgroup analyses, the differences in mean PI and RI values after haze exposure were pronounced in the symptomatic group; (3) Participants who reported new psychosomatic symptoms during haze exposure developed a significant decrease in PI and RI on TCD while the asymptomatic volunteers did not show any significant change in these TCD parameters. We postulate that participants could have had different susceptibility to the vasoactive effect of haze constituents on their cerebral vasculature.

TCD is a non-invasive bed-side tool that enables a reliable assessment of cerebral hemodynamics. It allows continuous monitoring of the blood flow in major intracranial arteries, which allows the evaluation of the impact of various vasoactive stimuli and challenges. For example, MFV and PI have been used in traumatic brain injury and subarachnoid hemorrhage as a surrogate measure of cerebral vasospasm and raised intracranial pressure, respectively.[23–25] PI represents the inherent resistance to the blood flow in an artery, influenced by the nature and stiffness of the brain vessels as well as compliance of the surrounding brain parenchyma.[26] PI may be reduced if the vascular bed distal to the site of insonation develops vasodilation; increased carbon-dioxide being the strongest trigger.[18] RI is another TCD parameter sometimes used to assess the flow resistance, it represents flow resistance distal to the site of insonation.[18] A decrease in PI and RI represents cerebral vasodilation due to the ambient haze exposure. These findings were most prominent after haze exposure amongst the individuals who reported new symptoms. The most common symptoms experienced were headache and lethargy, where changes in cerebral hemodynamics appear to play an important role. In general, both PI and RI represent similar information about the vascular resistance distal to the site of TCD insonation and are often used interchangeably.

Our findings suggest that exposure to short-term haze air, containing higher amount of carbon-dioxide and other vasoactive particulate matter, resulted in mild cerebral vasodilation. Similar reductions in the peripheral resistance have been demonstrated in the form of changes in brachial artery diameter, flow and elasticity.[27, 28] Cerebral blood flow is regulated, in part by the release of nitric oxide from vascular endothelial cells, and could be altered by levels and fluctuations of arterial carbon dioxide concentrations by a similar mechanism.[29, 30] Although we found a relationship between the level of haze and PI and RI, there was no significant change in BHI. Perhaps, this observation occurred due to our subjects being young and healthy. The changes in cerebral hemodynamics could have been more pronounced if we had included elderly individuals with various cardiovascular risk factors.[10] Similarly, no significant change in CVRi was found. This may also be attributed to the relatively young study population without cardiovascular risk factors, hence their autonomic nervous system would likely be able to maintain cerebrovascular resistance through various hemodynamic adjustments.[31] Lastly, the result could be a statistical error given the small sample size.

In addition to the TCD parameters, we report a statistically significant increase in diastolic blood pressure and heart rate after haze exposure. These may represent physiological changes in response to short-term haze exposure, which have higher concentrations of carbon dioxide. Peripheral and central chemoreceptors are sensitive to increases in arterial partial pressure of carbon dioxide. When these chemoreceptors are activated, they trigger an increase in heart rate, cardiac output and blood pressure via complex neural-hormonal pathways.[32, 33]

Certain limitations of our study need to be acknowledged. During the study period, the Ministry of Environment did not release the data for hourly PM2.5 sub-index, PM10 sub-index, Sulphur Dioxide sub-index, O3 sub-index, Carbon Monoxide sub-index and Nitrogen Dioxide. Thus, it is an inherent limitation of our study that we are unable to identify the precise constituents of the haze air that is responsible for the maximal hemodynamic effect on the cerebral vasculature. In addition, the exact period of haze exposure for each participant could not be standardised, which may result in exposure to different constituents of the haze or different past exposure to haze prior to the experiment. Nonetheless, this opportunistic study was performed to evaluate the real-world effects of cerebral hemodynamic changes from exposure to haze in its entirety, considering it to be vasoactive cumulatively. Importantly, our findings support a larger study with analyses of the concentration of the individual constituents of haze. We had initially planned to have subsequent air samples analysed for the concentrations of its specific constituents. However, due to the political efforts by various local and international government agencies, there was no opportunity to enable the planned study as there was no recurrence of a haze crisis of that magnitude in the region thereafter. Second, our prospective study focused on investigating the development of symptoms after short term haze exposure in healthy subjects. Our findings may not be extrapolated to older patients and with significant comorbidities like diabetes, hypertension and steno-occlusive disease of cervico-cranial large arteries etc, which may further exacerbate the harmful effects of haze exposure. Third, while we demonstrate the possible association between acute symptoms and cerebral hemodynamic responses, the long-term implications of such association remain unknown. Fourth, differences in the nature of particulate matter at different geographic locations may also mean that the findings of our study may not be generalizable to air pollution from other geographic locations. Lastly, the contents of haze caused by the annual forest fires in Southeast Asia may not be similar to the contents of polluted air. We did not record the information on the levels of particulate material of 2.5 micron or lower (PM_2.5_), considered to be vasoactive and responsible for many of the long-term somatic effects of polluted air. [34–36]

## Conclusions

Our study provides preliminary estimates of the effects of haze on cerebral hemodynamic parameters, which are probably associated with various psychosomatic symptoms. Even a short-term exposure to only moderately unhealthy haze conditions induced these changes in susceptible individuals. Perhaps, a larger study may determine the short-term impact of haze on individual work efficiency as well as long-term health effects of cerebral hemodynamic changes.

## Supporting information

S1 Fig Subgroup analyses showing mean middle cerebral artery pulsatility index (PI) change after haze exposure. (JPG)

S2 Fig Subgroup analyses showing mean middle cerebral artery resistivity index (RI) change after haze exposure. (JPG)

S1 File De-identified database. (XLSX)

## References

[1] Goswami K, Choudhury HK, Saikia J. Factors influencing farmers’ adoption of slash and burn agriculture in North East India. Forest policy and economics. 2012;15:146–51.

[2] Secretariat ASEAN. Media release: 6th meeting of the conference of the parties to the ASEAN agreement on transboundary. Jakarta: Association of Southeast Asian Nations; 2010. http://www.asean.org/storage/images/2012/publications/ASEAN%20DOCUMENTS%20SERIES%202010.pdf

[3] Quah E, Johnston D. Forest fires and environmental haze in Southeast Asia: using the ‘stakeholder’approach to assign costs and responsibilities. J Environ Manage. 2001;63(2):181–91. 10.1006/jema.2001.0475 11721597

[4] Tacconi L. Forest fire in Indonesia: cause, cost, policy implication. CIFOR Occasional Paper. 2003;(38).

[5] O’Callaghan J. Singapore, Malaysia face economic hit from prolonged smog. Reuters 2013.

[6] Awang MB, Jaafar AB, Abdullah AM, Ismail MB, Hassan MN, Abdullah R, et al Air quality in Malaysia: impacts, management issues and future challenges. Respirology. 2000;5(2):183–96. 10894109 10.1046/j.1440-1843.2000.00248.x

[7] Long W, Tate RB, Neuman M, Manfreda J, Becker AB, Anthonisen NR. Respiratory symptoms in a susceptible population due to burning of agricultural residue. Chest. 1998;113(2):351–7. 9498951 10.1378/chest.113.2.351

[8] Tan WC, Qiu D, Liam BL, Ng TP, Lee SH, van EEDEN SF, et al The human bone marrow response to acute air pollution caused by forest fires. Am J Respir Crit Care Med. 2000;161(4):1213–7. 10764314 10.1164/ajrccm.161.4.9904084

[9] Auchincloss AH, Roux AVD, Dvonch JT, Brown PL, Barr RG, Daviglus ML, et al Associations between recent exposure to ambient fine particulate matter and blood pressure in the Multi-Ethnic Study of Atherosclerosis (MESA). Environ Health Perspect. 2008;116(4):486 10.1289/ehp.10899 18414631 PMC2291007

[10] Wellenius GA, Boyle LD, Wilker EH, Sorond FA, Coull BA, Koutrakis P, et al Ambient fine particulate matter alters cerebral hemodynamics in the elderly. Stroke. 2013;44(6):1532–6. 10.1161/STROKEAHA.111.000395 23709640 PMC3722046

[11] Ho RC, Zhang MW, Ho CS, Pan F, Lu Y, Sharma VK. Impact of 2013 south Asian haze crisis: study of physical and psychological symptoms and perceived dangerousness of pollution level. BMC Psychiatry. 2014;14(1):81. 24642046 10.1186/1471-244X-14-81 PMC3995317

[12] Ramakreshnan L, Aghamohammadi N, Fong CS, Bulgiba A, Zaki RA, Wong LP, et al Haze and health impacts in ASEAN countries: a systematic review. Environmental Science and Pollution Research. 2017:1–16. 10.1007/s11356-017-0860-y 29209970

[13] Valentić D, Mićović V, Kolarić B, Brnčić N, Ljubotina A. The role of air quality in perception of health of the local population. Coll Antropol. 2010;34(2):113–7. 21305731

[14] Rockman CB, Svahn JK, Willis DJ, Lamparello PJ, Adelman MA, Jacobowitz GR, et al Carotid endarterectomy in patients 55 years of age and younger. Ann Vasc Surg. 2001;15(5):557–62. 10.1007/s10016-001-0029-4 11665441

[15] Alexandrov AV, Sloan MA, Wong LK, Douville C, Razumovsky AY, Koroshetz WJ, et al Practice standards for transcranial Doppler ultrasound: part I–test performance. J Neuroimaging. 2007;17(1):11–8. 10.1111/j.1552-6569.2006.00088.x 17238867

[16] Gosling R, King D. The Role of Measurement in Peripheral Vascular Surgery: Arterial Assessment by Doppler-shift Ultrasound. SAGE Publications; 1974. 10.1177/00359157740676P113 PMC1645777 4850636

[17] Pourcelot L. Diagnostic ultrasound for cerebral vascular disease. Present and Future Diagnostic Ultrasound. 1973:141–7.

[18] Markus H, Harrison M. Estimation of cerebrovascular reactivity using transcranial Doppler, including the use of breath-holding as the vasodilatory stimulus. Stroke. 1992;23(5):668–73. 1579964 10.1161/01.str.23.5.668

[19] Sharma AK, Bathala L, Batra A, Mehndiratta MM, Sharma VK. Transcranial Doppler: Techniques and advanced applications: Part 2. Annals of Indian Academy of Neurology. 2016;19(1):102 10.4103/0972-2327.173407 27011639 PMC4782524

[20] Ostermann K, Brauer M. Air quality during haze episodes and its impact on health Forest Fires and Regional Haze in Southeast Asia, ed by Eaton P, and Radojevic M. 2001;2.

[21] Pinto J, Grant L, Hartlage T. Report on USEPA air monitoring of haze from SE Asia biomass fires. Research Triangle Park, NC (National Centre for Environmental Assessment-RTP Office). Report No. EPA/600/R-98/071, USA, 1998.

[22] Agency NE. ‘Why is NEA’s Air Quality Data Different from the real-time data provided by other websites, such as aqicn.org? Is NEA’s data inaccurate?' 2017 [9 Aug 2018]. https://va.ecitizen.gov.sg/CFP/CustomerPages/NEA_google/displayresult.aspx?MesId=1543232&Source=Google&url=va.ecitizen.gov.sg

[23] McQuire JC, Sutcliffe JC, Coats TJ. Early changes in middle cerebral artery blood flow velocity after head injury. J Neurosurg. 1998;89(4):526–32. 10.3171/jns.1998.89.4.0526 9761044

[24] Moreno JA, Mesalles E, Gener J, Tomasa A, Ley A, Roca J, et al Evaluating the outcome of severe head injury with transcranial Doppler ultrasonography. Neurosurg Focus. 2000;8(1):1–7. 10.3171/foc.2000.8.1.1702 16906703

[25] Romner B, Bellner J, Kongstad P, Sjöholm H. Elevated transcranial Doppler flow velocities after severe head injury: cerebral vasospasm or hyperemia? J Neurosurg. 1996;85(1):90–7. 10.3171/jns.1996.85.1.0090 8683288

[26] Ursino M, Giulioni M, Lodi CA. Relationships among cerebral perfusion pressure, autoregulation, and transcranial Doppler waveform: a modeling study. J Neurosurg. 1998;89(2):255–66. 10.3171/jns.1998.89.2.0255 9688121

[27] Brook RD, Brook JR, Urch B, Vincent R, Rajagopalan S, Silverman F. Inhalation of fine particulate air pollution and ozone causes acute arterial vasoconstriction in healthy adults. Circulation. 2002;105(13):1534–6. 11927516 10.1161/01.cir.0000013838.94747.64

[28] O’Neill MS, Veves A, Zanobetti A, Sarnat JA, Gold DR, Economides PA, et al Diabetes enhances vulnerability to particulate air pollution–associated impairment in vascular reactivity and endothelial function. Circulation. 2005;111(22):2913–20. 10.1161/CIRCULATIONAHA.104.517110 15927967

[29] Lavi S, Gaitini D, Milloul V, Jacob G. Impaired cerebral CO2 vasoreactivity: association with endothelial dysfunction. American Journal of Physiology-Heart and Circulatory Physiology. 2006;291(4):H1856–H61. 10.1152/ajpheart.00014.2006 16766649

[30] White RP, Hindley C, Bloomfield PM, Cunningham VJ, Vallance P, Brooks DJ, et al The effect of the nitric oxide synthase inhibitor L-NMMA on basal CBF and vasoneuronal coupling in man: a PET study. J Cereb Blood Flow Metab. 1999;19(6):673–8. 10.1097/00004647-199906000-00011 10366198

[31] Robertson AD. Cerebrovascular hemodynamics in older adults: Associations with lifestyle, peripheral vascular health and functional decline. 2013.

[32] Barrett KE, Barman SM, Boitano S, Brooks H. Ganong’s review of medical physiology. 23 NY: McGraw-Hill Medical 2009.

[33] Guyenet PG. The sympathetic control of blood pressure. Nature Reviews Neuroscience. 2006;7(5):335 10.1038/nrn1902 16760914

[34] Miller KA, Siscovick DS, Sheppard L, Shepherd K, Sullivan JH, Anderson GL, et al Long-term exposure to air pollution and incidence of cardiovascular events in women. N Engl J Med. 2007;356(5):447–58. 10.1056/NEJMoa054409 17267905

[35] Wellenius GA, Burger MR, Coull BA, Schwartz J, Suh HH, Koutrakis P, et al Ambient air pollution and the risk of acute ischemic stroke. Arch Intern Med. 2012;172(3):229–34. 10.1001/archinternmed.2011.732 22332153 PMC3639313

[36] Weuve J, Puett RC, Schwartz J, Yanosky JD, Laden F, Grodstein F. Exposure to particulate air pollution and cognitive decline in older women. Arch Intern Med. 2012;172(3):219–27. 10.1001/archinternmed.2011.683 22332151 PMC3622279

