# Supplementary figures and images for: Psychosomatic symptoms during South East Asian haze crisis are related to changes in cerebral hemodynamics
Source: PLoS One. 2019 Jan 7;14(1):e0208724. doi: 10.1371/journal.pone.0208724 (PMC6322770; doi:10.1371/journal.pone.0208724)

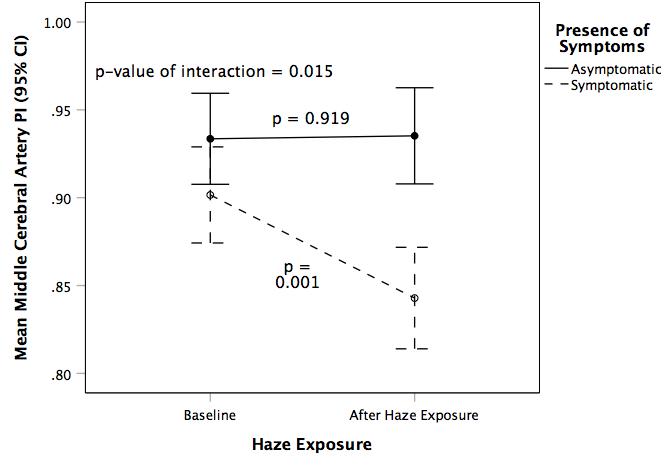

Supplement: S1 Fig — (JPG) [file pone.0208724.s001.jpg]

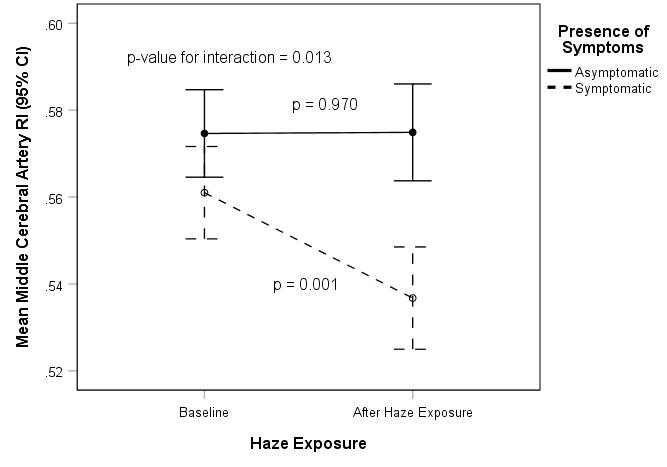

Supplement: S2 Fig — (JPG) [file pone.0208724.s002.jpg]
